# 2,6-DMBQ is a novel mTOR inhibitor that reduces gastric cancer growth in vitro and in vivo

**DOI:** 10.1186/s13046-020-01608-9

**Published:** 2020-06-09

**Authors:** Xueyin Zu, Xiaoli Ma, Xiaomeng Xie, Bingbing Lu, Kyle Laster, Kangdong Liu, Zigang Dong, Dong Joon Kim

**Affiliations:** 1 The Pathophysiology Department, The School of Basic Medical Sciences, Zhengzhou University, Zhengzhou, 450008 Henan China; 2 China-US (Henan) Hormel Cancer Institute, Zhengzhou, 450008 Henan China; 3 The Affiliated Cancer Hospital, Zhengzhou University, Zhengzhou, 450008 Henan China; 4 The Collaborative Innovation Center of Henan Province for Cancer Chemoprevention, Zhengzhou, 450008 Henan China; 5 International joint research center of cancer chemoprevention, Zhengzhou, China

**Keywords:** 2,6-DMBQ, mTOR, p70S6K, Gastric cancer, Patient-derived xenograft

## Abstract

**Background:**

Fermented wheat germ extract has been reported to exert various pharmacological activities, including anti-oxidant, anti-cell growth and cell apoptosis in various cancer cells. Although 2,6-dimethoxy-1,4-benzoquinone (2,6-DMBQ) is a benzoquinone compound and found in fermented wheat germ extract, its anticancer effects and molecular mechanism(s) against gastric cancer have not been investigated.

**Methods:**

Anticancer effects of 2,6-DMBQ were determined by MTT, soft agar, cell cycle and Annexin V analysis. Potential candidate proteins were screened via in vitro kinase assay and Western blotting. mTOR knockdown cell lines were established by lentiviral infection with shmTOR. The effect of 2,6-DMBQ on tumor growth was assessed using gastric cancer patient-derived xenograft models.

**Results:**

2,6-DMBQ significantly reduced cell growth and induced G1 phase cell cycle arrest and apoptosis in gastric cancer cells. 2,6-DMBQ reduced the activity of mTOR in vitro. The inhibition of cell growth by 2,6-DMBQ is dependent upon the expression of the mTOR protein. Remarkably, 2,6-DMBQ strongly reduced patient-derived xenograft gastric tumor growth in an in vivo mouse model.

**Conclusions:**

2,6-DMBQ is an mTOR inhibitor that can be useful for treating gastric cancer. It has therapeutic implications for gastric cancer patients.

## Background

Gastric cancer (GC) is a cancer of the digestive tract that remains one of the common malignant cancers worldwide [1, 2]. Specifically, it is the third leading cause of cancer-related mortality and the second frequently diagnosed cancer in the world [3]. Although clinical advances have been made in the fields of surgery, radiotherapy, and chemotherapy, the five-year survival rate of gastric cancer patients is approximately 15 to 35% [4]. Additionally, many types of targeted therapies, including inhibition of tyrosine kinase (TK) and receptor tyrosine kinase (RTK), are currently being used as treatment options for GC; however, they have shown only minimal efficacy [5, 6]. Therefore, identification of novel therapeutic targets and inhibitors are important for improving the survival rate of gastric cancer patients.

Mammalian target of rapamycin (mTOR) plays a central role in cell proliferation, cell motility, cell survival, cellular metabolism and protein synthesis [7]. mTOR is a serine/threonine protein kinase that is activated by various growth factors, cellular energy, cell stress and amino acids [8]. mTOR is classified structurally and functionally in two complexes, mTOR complex 1 (mTORC1) and mTOR complex 2 (mTORC2), which share common subunits, such as mTOR, the mammalian lethal with SEC13 protein 8 (mLST8), telomere maintenance 2 (Tel2) and Tel2-interacting protein 1 (Tti1) [9]. mTORC1 contains the regulatory-associated protein of mTOR (RAPTOR), which is a scaffolding protein in the mTORC1 assembly, and mTORC2 contains the rapamycin-insensitive companion of mTOR (RICTOR) [10]. AKT phosphorylates Ser2448 of mTOR in addition to tuberous sclerosis complex 2 (TSC2) thereby resulting in activation of mTOR kinase activity [11]. Additionally, mTOR is auto-phosphorylated at Ser2481 which is located in a hydrophobic region near the conserved carboxyl-terminal and required for FRAP kinase activity [12]. The complex in which it participates dictates the substrate specificity of mTOR. The mTORC1 substrate S6 Kinase 1 (S6K1) associates with mRNAs and regulates both mRNA translation initiation and progression, thus enhancing protein synthesis [13]. S6K1 is a serine/threonine protein kinase that is necessary for cell growth and G1 cell cycle progression [14]. In contrast, mTORC2 phosphorylates and activates v-Akt murine thymoma viral oncogene homolog (AKT) which regulates cell growth, cell survival and cell cycle progression [13]. AKT is a serine/threonine kinase that belongs to the AGC family of kinases [15], and regulates many cellular functions, including proliferation, survival, epithelial mesenchymal transition (EMT), and metabolism; additionally, AKT directly phosphorylates a wide range of downstream substrates [16]. mTOR is dysregulated in various cancers due to its direct mutation, mutations of mTOR components and mutation of upstream genes including oncogenes and tumor suppressor genes [17]. mTOR and AKT are overexpressed in GC cells and the mTOR pathway is activated in 60% of GC patients [18]. Currently, mTOR inhibitors have been investigated in preclinical studies and clinical trials of GC [19]. mTOR inhibitors have been tested in many clinical trials in the context of other cancers, but they achieved only modest efficacy applied as monotherapies in cancer treatments due to resistance mechanisms [20, 21]. Therefore, combined therapies with mTOR inhibitors and other target inhibitors are under investigation in preclinical and clinical trials in various cancers [22]. Thus, novel therapeutic strategies with mTOR inhibitor should be further investigated.

Fermented wheat germ extract possesses preventive and therapeutic functions in various cancer cells [23, 24]. 2,6-Dimethoxy-1,4-benzoquinone (2,6-DMBQ), a derivative of fermented wheat germ extract, is found in sourdough fermentation of wheat germ and other fermented foods. However, the anticancer activity of 2,6-DMBQ and its molecular mechanism(s) against gastric cancer have not been investigated. In the present study, we report that 2,6-DMBQ is a novel mTOR inhibitor that reduces gastric cancer growth in vitro and in vivo.

## Methods

### Reagents and antibodies

2,6-DMBQ was purchased from Shanghai Chemic Industry (Shanghai, China). Dimethyl sulfoxide (DMSO) was purchased from Tianjin Kemai Chemical Reagent Company (Tianjin, China). AZD8055 was purchased from Selleckchem (Houston, TX, USA) and CMPD101 was purchased from MedChemExpress (Monmouth Junction, NJ, USA). RPMI 1640 medium and fetal bovine serum (FBS) were purchased from Biological Industries (Cromwell, CT, USA). MEM/EBSS medium was purchased from GE Healthcare (Logan, UT, USA). Active mTOR recombinant protein for kinase assay was purchased from ThermoFisher (Shanghai, China). Inactive p70S6K recombinant protein for in vitro kinase assay was purchased from SignalChem (Richmond, BC, Canada). The antibody to detect β-actin was from Santa Cruz Biotechnology (Santa Cruz, CA, USA), and all the other antibodies were purchased from Cell Signaling Technology (Beverly, MA, USA).

### Cell lines

AGS, HGC27, NCI-N87 and SNU-1 gastric cancer cells were obtained from the Cell Bank of the Chinese Academy of Sciences (Shanghai, China). JB6 mouse epithelial cells were purchased from American Type Culture Collection (Manassas, VA, USA). Enough frozen vials were available for each cell line to ensure that all cell-based experiments were conducted on cells that had been authenticated and in culture for a maximum of 8 weeks. AGS, NCI-N87 and SNU-1 cells were cultured in Roswell Park Memorial Institute medium 1640 (RPMI1640) medium with 10% FBS and 1% penicillin–streptomycin. HGC27 cells were cultured in Minimum Essential Medium with Earle’s Balanced Salts (MEM/EBSS) supplemented with 1% non-essential amino acid (NEAA), 10% FBS and 1% antibiotic-antimycotic. The JB6 cells were cultured in MEM supplemented with 5% FBS and 1% penicillin–streptomycin. All cells were maintained at 37 °C in a 5% CO2 humidified incubator.

### Cell proliferation assay

AGS (1.2 × 10^3^ cells per well) or HGC27 (2.0 × 10^3^ cells per well) cells were seeded in 96-well plates with 100 μl complete growth medium (10% FBS) and incubated for 24 h. Cells were treated with various concentrations of 2,6-DMBQ (dissolved in DMSO) or vehicle (DMSO) in 100 μl of complete growth medium. After incubation for 48 h, 20 μl of the MTT solution (Solarbio, Beijing, China) were added to each well. After incubation for 2 h at 37 °C in a 5% CO_2_ incubator, the cell culture medium was removed. Subsequently, 150 μl of DMSO was added to each well and the crystal formation was dissolved. Absorbance was measured at 570 nm using the Thermo Multiskan plate-reader (Thermo Fisher Scientific, Waltham, MA, USA).

### Anchorage-independent cell growth assay

Cells (8 × 10^3^ cells per well) suspended in complete growth medium supplemented with 10% FBS were added to 0.3% agar with different concentrations of 2,6-DMBQ (dissolved in DMSO) or vehicle (DMSO) in a top layer over a base layer of 0.6% agar with or without different concentrations of 2,6-DMBQ. The cultures were maintained at 37 °C in a 5% CO_2_ incubator for 2 weeks and then colonies were imaged under a microscope and quantified using the Image-Pro Plus software (v.6) program (Media Cybernetics, Rockville, MD, USA).

### Western blot analysis

Cells were lysed in radio-immunoprecipitation assay buffer (RIPA) buffer (50 mM Tris-HCl pH 7.4, 1% NP-40, 0.25% sodium deoxycholate, 0.1% sodium dodecyl sulfate, 150 mM NaCl, 1 mM EDTA, 1 × protease inhibitor solution), and incubated on ice for 1 h. The soluble cell lysates were collected by centrifugation at 10000 g for 10 min. Proteins were separated by SDS/PAGE and transferred to polyvinylidene difluoride membranes (Amersham Biosciences, Piscataway, NJ, USA). Membranes were blocked with 5% nonfat dry milk at room temperature for 1 h and incubated with appropriate primary antibodies at 4 °C for overnight. The next day the membranes were washed with TBST, followed by 1 h incubation with 1:5000 dilution of horseradish peroxidase–linked secondary antibody. The immuno-reactive proteins were detected by chemiluminescence reagent (Amersham Biosciences Corp) using the ImageQuant LA S4000 system (GE Healthcare, Piscataway, NJ, USA).

### In vitro ATP assay for mTOR kinase activity

To determine mTOR kinase activity, an ATP assay was carried out using the ADP-Glo Kinase Assay Kit, in accordance with the manufacturer’s instructions (Promega, Madison, WI, USA). The active recombinant mTOR (50 ng) protein was mixed with different concentrations of 2,6-DMBQ, AZD8055 (dissolved in DMSO) as a mTOR inhibitor, or vehicle (DMSO) in reaction buffer (Cell Signaling Technology) and incubated at room temperature for 15 min. The inactive p70S6K recombinant protein (100 ng) and ATP were added and the mixtures were incubated at 30 °C for 30 min. The fluorescence of each sample was measured at excitation and emission wavelengths of 530 nm and 590 nm, respectively.

### Cell cycle analysis

AGS (6 × 10^4^ cells per dish) or HGC27 (7 × 10^4^ cells per dish) cells were plated into 60-mm culture dishes and incubated for 24 h. Cells were synchronized by serum starvation for 24 h and treated with serum and 2,6-DMBQ (dissolved in DMSO) or vehicle (DMSO) for 24 h in 10% serum and medium. Cells were collected by trypsinization and washed with phosphate-buffered saline (PBS) and then fixed in 1000 μl of 70% cold ethanol. After rehydration, cells were incubated in RNase (100 μg/mL) and stained with propidium iodide (PI; 20 μg/mL). PI staining was accomplished following the manufacturer’s instructions (Clontech, Palo Alto, CA) and the cells were analyzed by flow cytometry.

### Apoptosis assay

Cells were plated into 6 well plates (5 × 10^4^ cells per well). After incubation for 24 h, cells were treated with different doses of 2,6-DMBQ (dissolved in DMSO) or vehicle (DMSO) for 48 h in 10% serum-containing medium. Cells were collected by trypsinization and washed with PBS. Cells were subsequently stained with Annexin V (BioLegend, San Diego, CA) and propidium iodide before apoptosis was analyzed by flow cytometry.

### Lentiviral infection

Short hairpin RNA sequences against mTOR were designed (#3, 5′-CCGGCCCGGATCATTCACCCTATTGCTCGAGCAATAGGGTGAATGA.

TCCGGGTTTTTG-3′; #4, 5′-CCGGGAACCAATTATACCCGTTCTTCTCGAGAA.

GAACGGGTATAATTGGTTCTTTTTG-3′) and cloned into the lentiviral vector (pLKO.1-mTOR). The lentiviral packaging vectors (pMD2.0G and psPAX) were purchased from Addgene Inc. (Cambridge, MA, USA). To prepare mTOR viral particles, each viral vector and package vectors were transfected into HEK293T cells by using Lipofectamine 2000 (Invitrogen, Grand Island, NY, USA) following the manufacturer’s suggested protocol. After incubation for 48 h, viral particles were harvested by filtration using a 0.45 mm sodium acetate syringe filter. The virus-containing media was combined with 8 μg/ml of polybrene (Millipore, Billerica, MA, USA) before being used to infect AGS or HGC27 cells. After incubation for 24 h, cells were selected with puromycin (1 μg/ml) for 48 h. The selected cells were used for experiments.

### Patient-derived xenograft gastric tumor growth assay and ethics statement

To examine the effect of 2, 6-DMBQ on patient-derived gastric tumor growth, female mice (Vital River Labs, Beijing, China) with severe combined immunodeficiency (SCID; 6–9 weeks old) were maintained under “specific pathogen-free” conditions based on the guidelines established by Zhengzhou University Institutional Animal Care and Use Committee (Zhengzhou, China). Human tumor specimens of gastric cancer tissue were obtained from the Affiliated Cancer Hospital in Zhengzhou University. The gastric cancer patients did not receive any chemotherapy or radiotherapy prior to surgery. Tissue histology was confirmed by a pathologist. Prior written informed consent was obtained from patients. Mice were anesthetized by 0.4% pentobarbital sodium (Sinopharm Chemical Reagent Co., Ltd., Shanghai, China). Mice were pierced to the back of neck using a blunt puncher and then treated with Penicillin-Streptomycin (80,000 U/ml) in the affected area. Gastric cancer tissues composed of normal, cancerous stromal and tumor were cut into pieces (3–4 mm^3^) and implanted into the back of the neck of 3 individual mice. After the 3rd generation of human gastric cancer tissue growth, tissues were again cut into pieces and implanted into mice. Mice were divided into 2 groups of 7 animals as follows: 1) vehicle (10% DMSO and 20% tween 80) group and 2) 80 mg 2,6-DMBQ/kg of body weight in vehicle (10% DMSO and 20% tween 80) were administered by oral gavage once a day Monday through Friday. Tumor volume was calculated from measurements of 2 diameters of the individual tumor base using the following formula: tumor volume (mm^3^) = (length ×width× height× 0.52). Mice were monitored until tumors reached 1.5cm^3^ total volume, at which time mice were euthanized and tumors, liver, kidney, and spleen extracted.

### Hematoxylin-eosin staining and immunohistochemistry

The liver, spleen, kidney, and tumor tissues from mice were embedded in paraffin blocks and used for hematoxylin and eosin (H&E) staining or immunohistochemistry (IHC). For H&E staining, the tissue sections were deparaffinized, hydrated and stained with H&E and then dehydrated. For IHC, tumor tissue sections were deparaffinized and hydrated. After antigen retrieval with 10 mM citrate acid and blocking with 5% BSA, the tumor tissue sections were hybridized with a primary antibody (Ki-67, 1:100; Thermo Fisher Scientific) for 18 h at 4 °C and then an HRP-conjugated goat anti-rabbit or mouse IgG antibody (ZSGB-BIO, Beijing, China) was added and incubated for 30 min. Tissue sections were developed with 3, 3′-diaminobenzidine (ZSGB-BIO) for 10 s and then counterstained with hematoxylin for 1 min. All sections were observed by microscope and analyzed using the Image-Pro Plus software (v. 6) program.

### In vivo toxicity assay

Female mice (SCID; 6–9 weeks old) were maintained under “specific pathogen-free” conditions based on the guidelines established by Zhengzhou University Institutional Animal Care and Use Committee. Mice were divided into 4 groups as follows: 1) vehicle group (n = 4); 2) 20 mg 2,6-DMBQ/kg of body weight in vehicle (n = 4); 3) 50 mg 2,6-DMBQ/kg of body weight in vehicle (n = 4); and 4) 80 mg 2,6-DMBQ/kg of body weight in vehicle (n = 4). 2,6-DMBQ or vehicle (10% DMSO in 20% tween 80) was orally administered for 2 weeks. Blood samples from each group of mice were collected in heparin-treated tubes. The AST or ALT activity from serum was measured at 510 nm.

### Statistical analysis

All quantitative results are expressed as mean ± S.D. or ± S. E values. Significant differences were compared using the Student’s t-test or one-way analysis of variance (ANOVA). Differences with a *p* < 0.05 were considered to be statistically significant. The statistical package for social science for Windows (IBM, Inc. Armonk, NY, USA) was used to calculate the p-value to determine statistical significance.

## Results

### 2,6-DMBQ reduces cell growth and induces G1 phase cell cycle arrest in gastric cancer cells

**Fig. 1:**
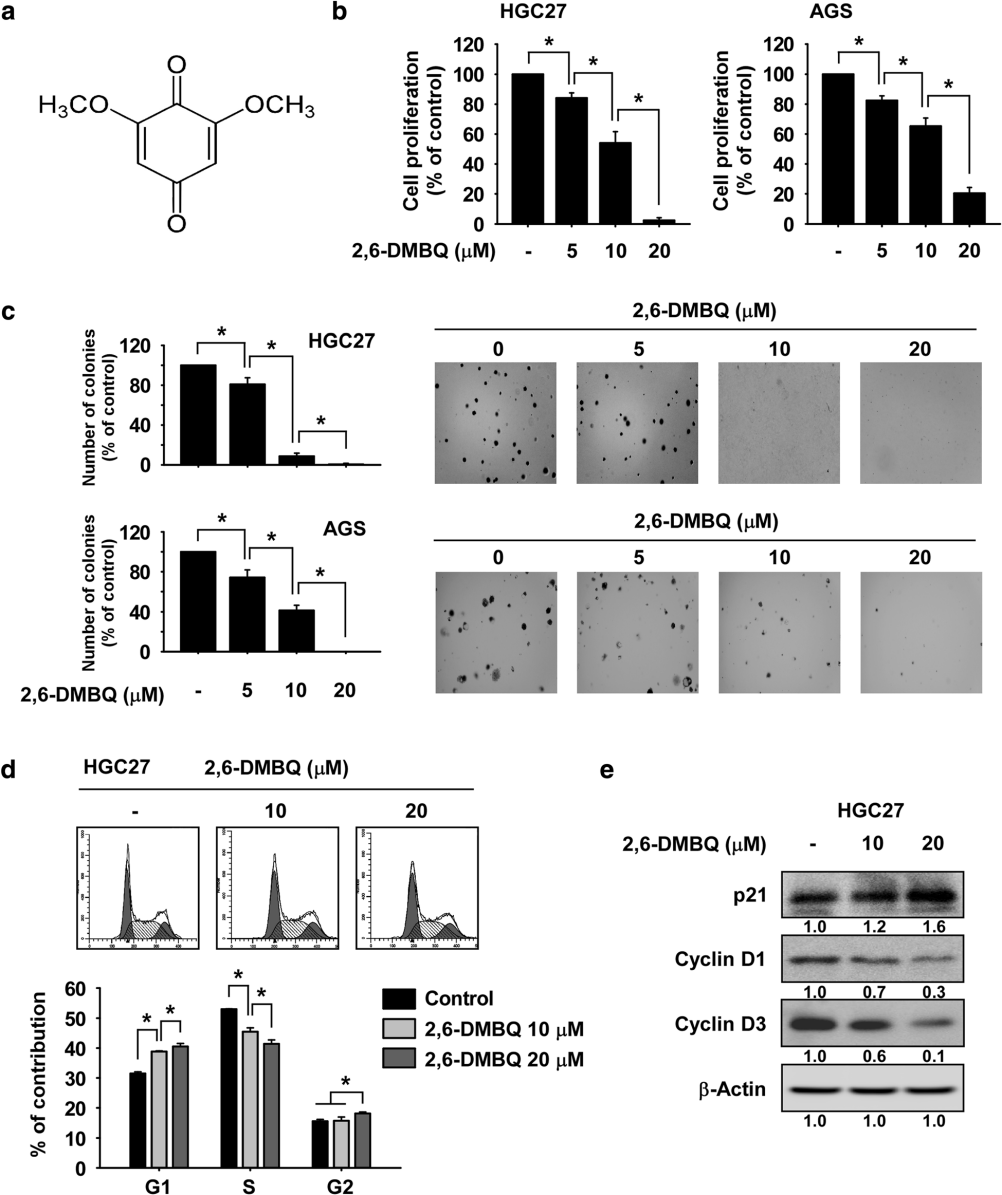
2,6-DMBQ reduces growth of gastric cancer cells. **a** Chemical structure of 2,6-DMBQ. **b** Effect of 2,6-DMBQ on growth of gastric cancer cells. Cells were treated with 2,6-DMBQ at various concentrations and then incubated for 48 h and growth was determined by the MTT assay. **c** Effect of 2,6-DMBQ on anchorage-independent growth of gastric cancer cells. Cells were treated with 2,6-DMBQ and incubated for 2 weeks and then colonies were counted using a microscope and the Image-Pro PLUS (v.6) computer software program. **d** Effect of 2,6-DMBQ on cell cycle. HGC27 gastric cancer cells were synchronized by serum starvation for 24 h and treated with 2,6-DMBQ for 24 h in 10% serum-supplemented medium. Cells were stained with propidium iodide (PI) and cell cycle was analyzed by Fluorescence Activated Cell Sorting (FACS). For **b**-**d**, data are shown as means ± S.D. of values from 3 independent experiments each with triplicate samples and the asterisk (*) indicates a significant (*p* < 0.05) difference. **e** Effect of 2,6-DMBQ on the expression of cell cycle marker proteins in HGC27 gastric cancer cells was determined by Western blotting. Band density was measured using the Image J (NIH) software program. For **e**, similar results were observed from 3 independent experiments

2,6-DMBQ is a 2,6-dimethoxy-1,4-benzoquinone that is classified as a benzoquinone compound (Fig. 1a). To examine the IC_50_ value of 2,6-DMBQ on growth of gastric cancer cells, HGC27 or AGS cells were treated with various concentrations of 2,6-DMBQ for 48 h. Results showed that 50% reduction of HGC27 or AGS cell growth was achieved at 10.1 μM or 18.7 μM, respectively (Supplemental Fig. 1a, b). Therefore, we used 2,6-DMBQ concentration at 5, 10 or 20 μM for the further study. To determine effect of 2,6-DMBQ on growth of gastric cancer cells, HGC27 or AGS cells were treated with 2,6-DMBQ for 48 h. The results indicated that growth of gastric cancer cells was reduced in a dose-dependent manner by 2,6-DMBQ treatment (Fig. 1b). We also examined the effect of 2,6-DMBQ on anchorage-independent growth of gastric cancer cells. The results showed that 2,6-DMBQ significantly reduced anchorage-independent growth of gastric cancer cells (Fig. 1c). Based on the anti-growth effect of 2,6-DMBQ, we next assessed whether 2, 6-DMBQ could affect cell cycle progression. HGC27 gastric cancer cells were synchronized by serum starvation for 24 h and treated with 2,6-DMBQ for 24 h in 10% serum and medium. Cell cycle was analyzed by flow cytometry and cell cycle marker proteins were detected by Western blotting. The results showed that 2,6-DMBQ strongly reduced the S phase fraction and induced G1 phase cell cycle arrest in a dose-dependent manner (Fig. 1d). Treatment with 2,6-DMBQ also increased the expression of p21, a marker protein of G1 phase, and decreased the expression of cyclin D1 and cyclin D3, marker proteins of S phase (Fig. 1e).

### 2,6-DMBQ induces apoptosis of gastric cancer cells

**Fig. 2:**
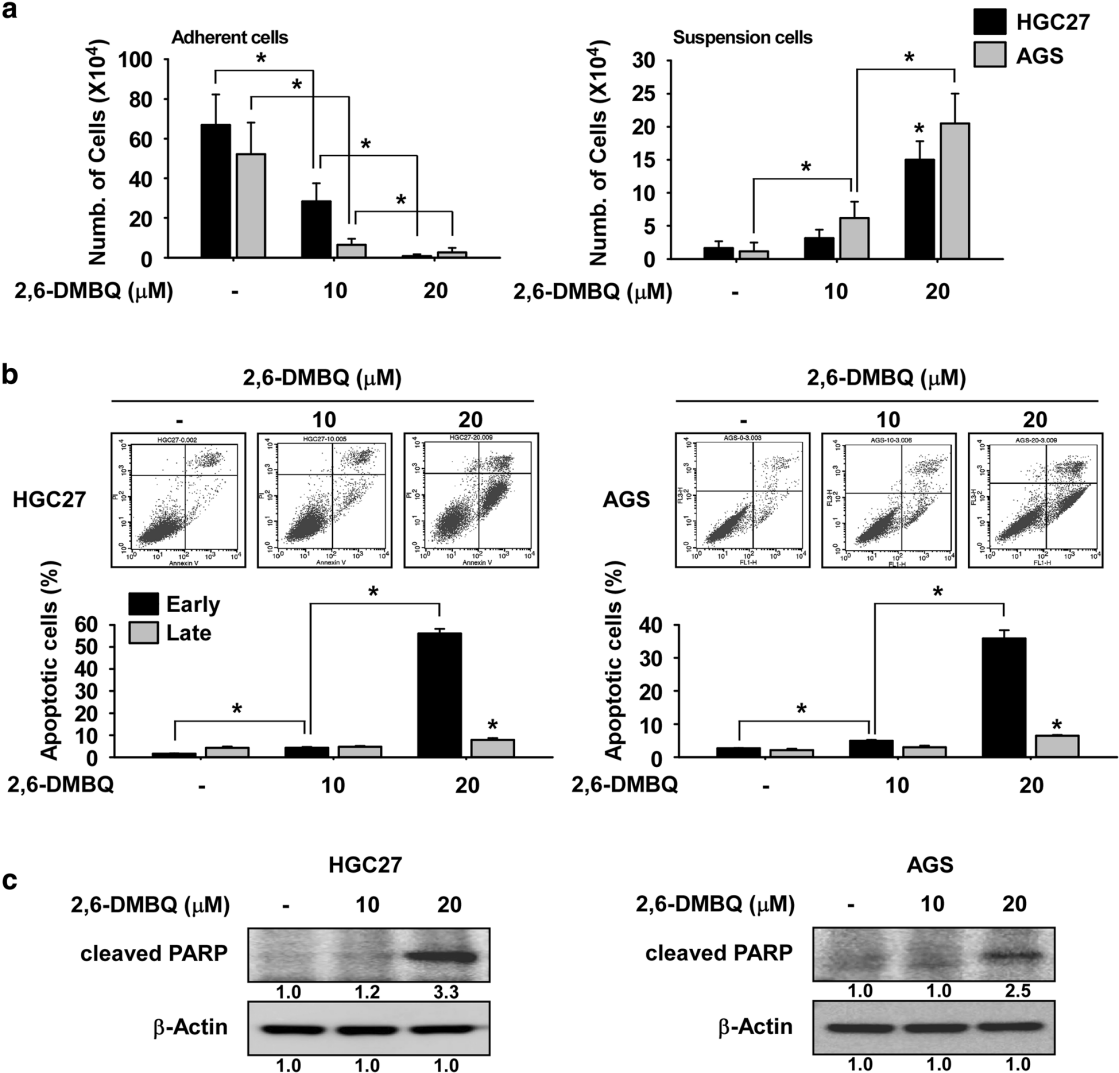
2,6-DMBQ induces apoptosis of gastric cancer cells. **a** Effect of 2,6-DMBQ on cancer cell death. Cells were seeded in a 6-well plate and treated with 2,6-DMBQ for 48 h. The number of suspended or attached cells was counted using a hematocytometer. **b** Effect of 2,6-DMBQ on cancer cell apoptosis. Cells were treated with 2,6-DMBQ in 10% FBS and then incubated for 48 h. Cells were stained with annexin V and propidium iodide (PI) and apoptosis was determined by Fluorescence Activated Cell Sorting (FACS). For **a**-**b**, data are shown as means ± S.D. of triplicate values from 3 independent experiments and the asterisk (*) indicates a significant (*p* < 0.05) difference. **c** Effect of 2,6-DMBQ on expression of proapoptotic marker protein. Cells were treated with 2,6-DMBQ for 48 h and the levels of cleaved PARP protein was determined by Western blotting. Band density was measured using the Image J (NIH) software program. For **c**, results of Western blotting are shown as mean values ± S.D. for 3 independent experiments

To investigate the effect of 2, 6-DMBQ on the programmed cell death of gastric cancer cells, we examined cell viability after treatment. AGS or HGC27 cells were treated with 2,6-DMBQ for 48 h and suspended cells (dead) and adherent cells (live) were counted. The results indicated that the number of suspended cells was significantly increased by 2, 6-DMBQ-treated cells compared with untreated control cells (Fig. 2a, *right panel*). In contrast, the number of adherent cells was significantly decreased with 2,6-DMBQ treatment (Fig. 2a, *left panel*). To determine whether 2,6-DMBQ-induced gastric cancer cell death was due to apoptosis, cells were treated with 2,6-DMBQ for 48 h and annexin V expression was analyzed. The results indicated that early apoptosis in 2,6-DMBQ-treated cells was significantly increased compared to untreated control cells (Fig. 2b). We also examined the effect of 2,6-DMBQ on apoptotic signaling pathways and the results showed that cleaved PARP was markedly increased (Fig. 2c).

### 2,6-DMBQ is a novel mTOR inhibitor

**Fig. 3:**
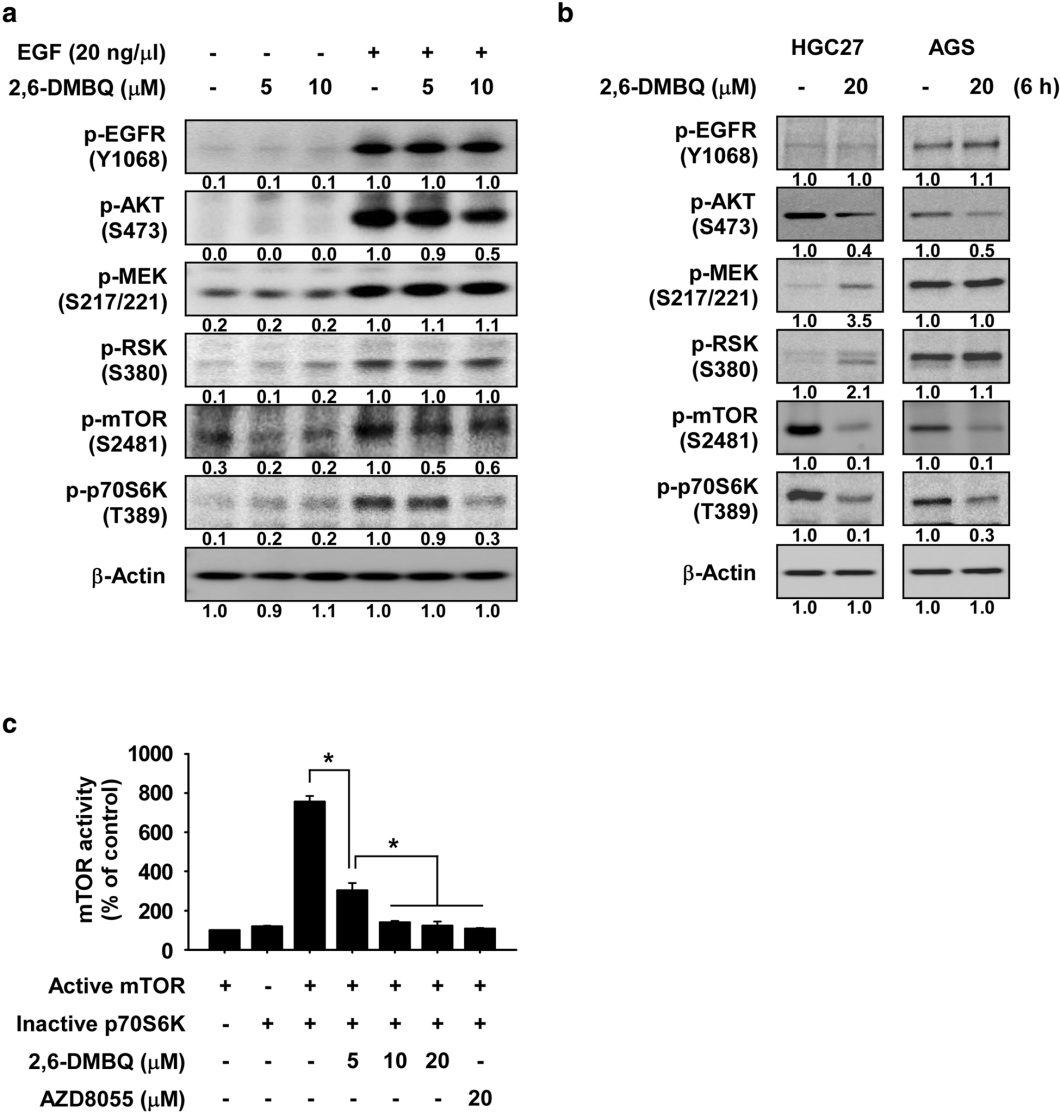
2,6-DMBQ strongly reduces the mTOR signaling pathway. **a** Effect of 2,6-DMBQ on EGF-induced kinase signaling in JB6 cells. Serum-starved (0.1% FBS; 24 h) cells were treated with different doses of 2,6-DMBQ for 2 h followed by treatment with EGF for 30 min. **b** Effect of 2,6-DMBQ on kinase signaling in gastric cancer cells. Cells were treated with 2,6-DMBQ and then various signaling proteins were examined by Western blotting. For **a** and **b**, band density was measured using the Image J (NIH) software program. All results of Western blotting are shown as mean values ± S.D. for 3 independent experiments. **c** Effect of 2,6-DMBQ on mTOR kinase activity. mTOR kinase activity was assessed by an in vitro kinase assay using active mTOR and inactive p70S6K proteins. mTOR inhibitor AZD8055 was used as a positive control. For **c**, data are shown as means ± S.D. of triplicate values from 3 independent experiments and the asterisk (*) indicates a significant (*p* < 0.05) difference

Previously, JB6 mouse epithelial cells have been used to identify potential molecular targets of selected compounds. Epidermal growth factor (EGF) can activate various signaling pathway [25]. Therefore, to identify potential molecular targets of 2,6-DMBQ, we first investigated whether 2,6-DMBQ could affect various EGF-induced signaling molecules in JB6 cells. After serum starvation for 24 h, cells were treated for 2 h with 2,6-DMBQ before addition of EGF for 0.5 h. Results showed that expression of phosphorylated AKT, mTOR and p70S6K was strongly reduced by 2,6-DMBQ, whereas other signaling proteins were not affected (Fig. 3a). We next assessed the effect of 2,6-DMBQ on various signaling pathways in gastric cancer cells after HGC27 and AGS cells were treated for 6 h with 2,6-DMBQ. The results indicated that the expression of phosphorylated AKT, mTOR and p70S6K were strongly decreased by 2,6-DMBQ, but had little effect on other signaling proteins (Fig. 3b). It is well reported that mTOR protein is a direct upstream kinase and phosphorylates AKT and p70S6K proteins [10]. Therefore, 2,6-DMBQ may attenuate AKT and p70S6K activation through reducing mTOR protein. Next, to examine whether 2,6-DMBQ could affect mTOR activity, we performed in vitro kinase assays using a recombinant active mTOR protein and an inactive p70S6K protein. The results indicated that 2,6-DMBQ reduced the phosphorylation of p70S6K in a dose-dependent manner by directly targeting mTOR (Fig. 3c). Furthermore, to investigate the other molecular target proteins of 2,6-DMBQ, 23 cancer-related kinases were screened by using a recombinant active kinase protein and the specific substrate for each kinase (Kinase profiling service, eurofins, https://www.eurofins.com). ABL, AMPKα1, AURKA, BRAF, CDK2/CCNE, CHEK1, EGFR, FAK, FGFR1, FYN, GSK3β, MAPK1, MEK1, MET, PDK1, PKBα, PKCα, p70S6K, RSK2, SAPK2α, SRC, TAK1, or TBK1 kinase and respective substrate were incubated with or without 2,6-DMBQ in an in vitro kinase assay. The results indicated that the activity of these kinases was not affected by 2,6-DMBQ (Supplemental Fig. 2).

### Knockdown of mTOR reduces gastric cancer cell growth

**Fig. 4:**
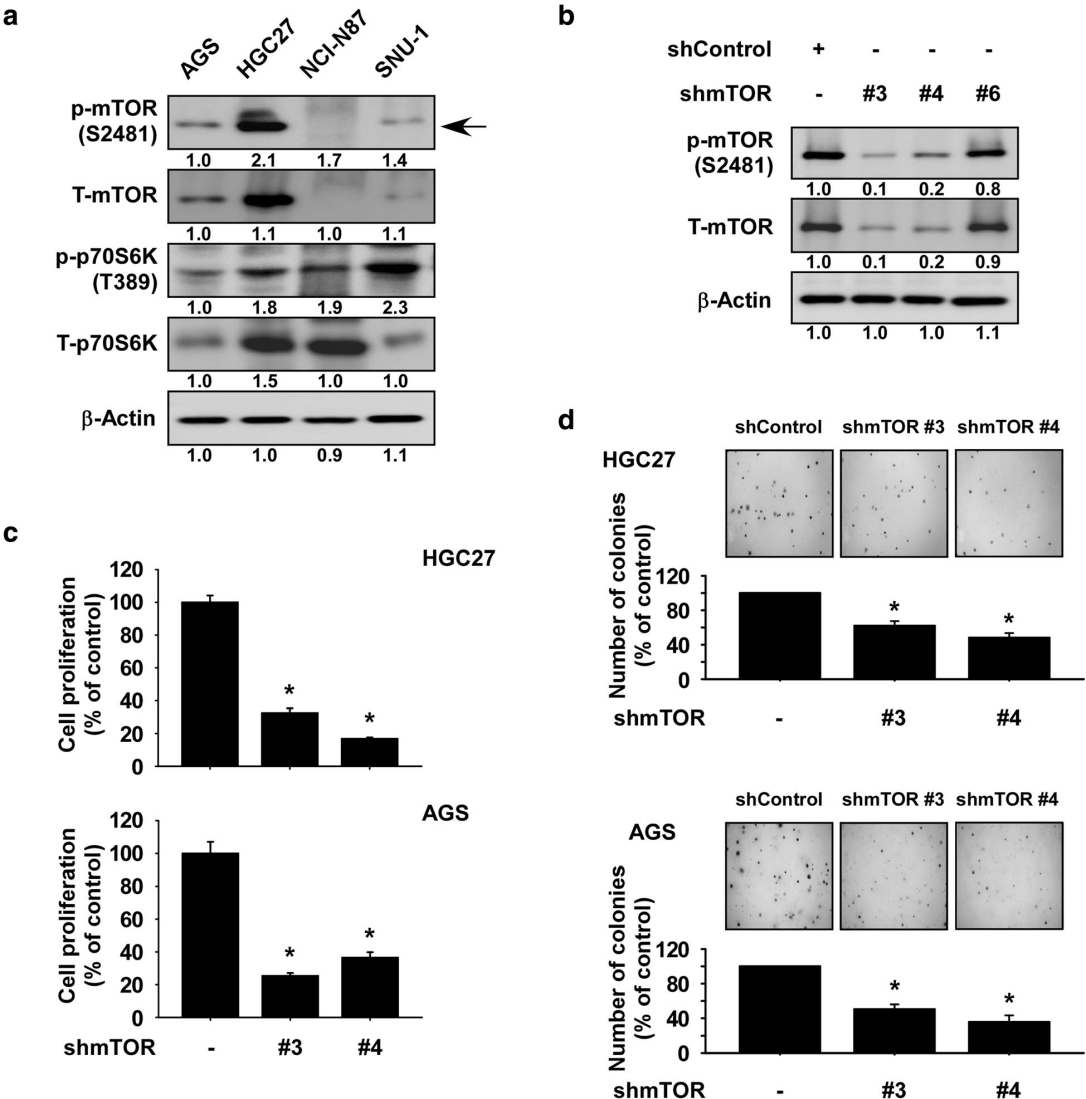
mTOR is a therapeutic target in gastric cancer cells. **a** Expression mTOR signaling molecules in gastric cancer cells. Cells were seeded and incubated for 48 h and expression of the total or phosphorylated mTOR and p70S6K proteins was analyzed by Western blotting. **b** Effect of mTOR knockdown on total or phosphorylated mTOR protein. HGC27 gastric cancer cells stably expressing knockdown mTOR or Control were established. The expression of total or phosphorylated mTOR was determined by Western blotting. For **a** and **b**, similar results were observed from 3 independent experiments and band density was measured using the Image J (NIH) software program. **c**, **d** Effect of mTOR knockdown on growth of gastric cancer cells. Cells were seeded and incubated for 48 h or 2 weeks and cell growth was determined by (**c**) MTT assay or (**d**) soft agar assay. For **e**, colonies were counted using a microscope and the Image-Pro PLUS (v.6) computer software program. For **d** and **e**, data are shown as means ± S.D. of triplicate values from 3 independent experiments. The asterisk (*) indicates a significant difference (*p* < 0.05)

To determine the levels of mTOR and p70S6K protein expression in gastric cancer cells, we performed Western blotting using 4 gastric cancer cell lines. The results showed that phosphorylated mTOR was highly expressed in AGS and HCG27 cells compared to NCI-N87 and SNU-1 cells (Fig. 4a); therefore, AGS and HGC27 cells were used for further cell-based studies. To determine the influence of mTOR knockdown on gastric cancer cell growth, we established cells stably expressing knockdown of mTOR or a control shRNA and determined the expression of mTOR protein by Western blotting. The results showed that expression of phosphorylated and total mTOR was strongly reduced in shmTOR #3 and shmTOR #4 cells (Fig. 4b). We next examined the effect of mTOR knockdown on anchorage-dependent or -independent growth of gastric cancer cells. Cells were seeded and incubated for 48 h or 2 weeks and cell growth was determined by MTT or soft agar assay, respectively. The results showed that anchorage-dependent and -independent growth of gastric cancer cells was significantly reduced by knockdown of mTOR (Fig. 4c, d).

### The reduction of cell growth by 2,6-DMBQ is dependent on the abundance of mTOR

**Fig. 5:**
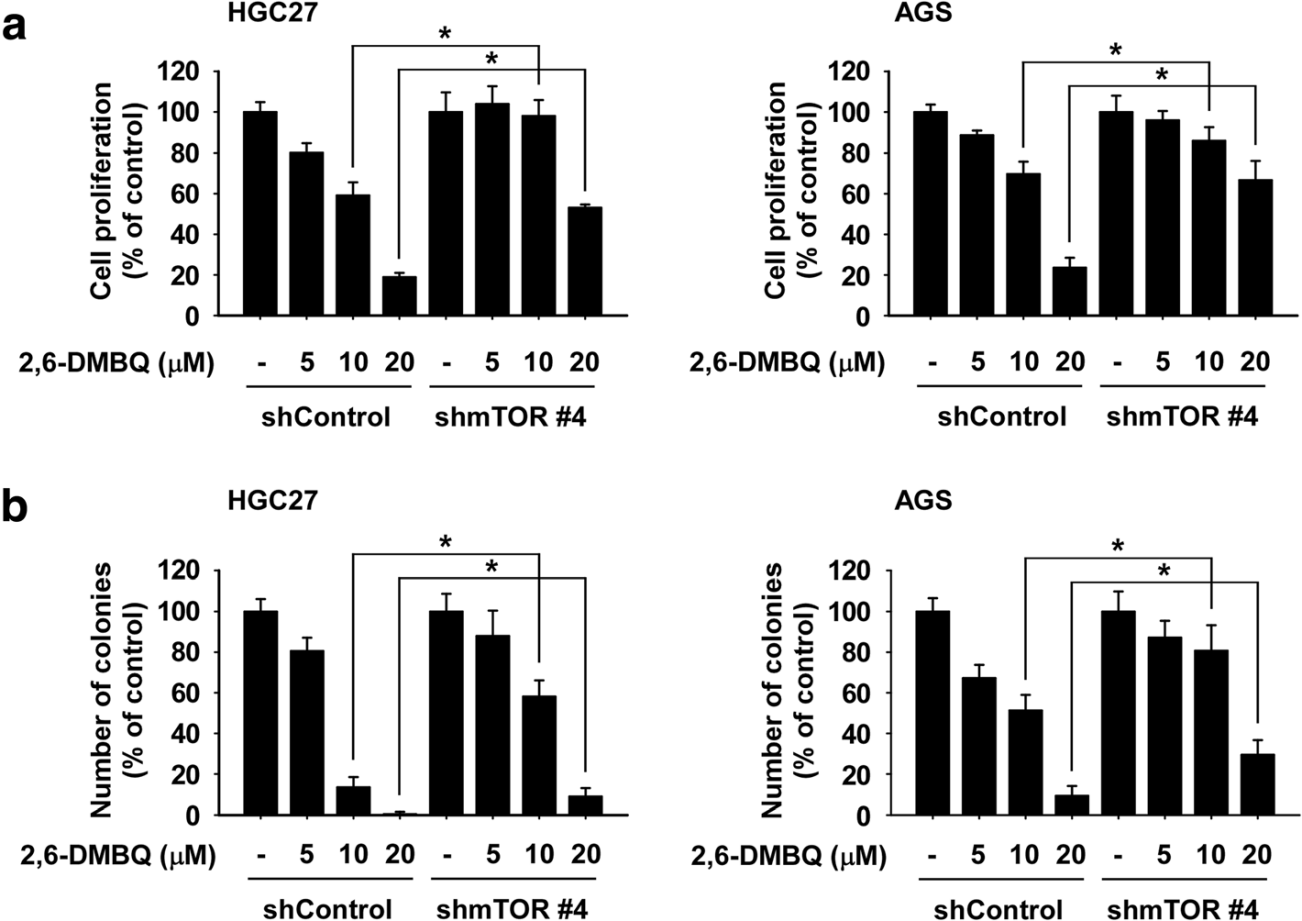
Reduction of cell growth by 2,6-DMBQ is dependent on the expression of mTOR. **a** The effect of 2,6-DMBQ on gastric cancer cell growth was assessed in cells stably expressing *shmTOR* or cells stably expressing *shControl*. Cells were seeded for 24 h and treated or not treated with 2,6-DMBQ at various concentrations and then incubated for 48 h and growth was determined by the MTT assay. **b** The effect of 2,6-DMBQ on anchorage-independent gastric cancer cell growth was assessed in cells stably expressing *shmTOR* or cells stably expressing *shControl*. Cells were treated with scutellarin and incubated for 2 weeks and then colonies were counted using a microscope and the Image-Pro PLUS (v.6) computer software program. All data are represented as means ± S.D. of triplicate values from 3 independent experiments. The asterisk (*) indicates a significant (*p* < 0.05) difference

To examine whether the reduction of gastric cancer cell growth by 2,6-DMBQ is dependent on the mTOR expression, cells expressing *shmTOR #4* or *shControl* were treated with 2,6-DMBQ for 48 h or 2 weeks. Anchorage-dependent or -independent growth of gastric cancer cells was determined by MTT or soft agar assay. The results indicated that cells expressing *shmTOR #4* were resistant to 2,6-DMBQ’s effect on cell growth compared to cells expressing *shControl* (Fig. 5a, b).

### 2,6-DMBQ reduces gastric cancer patient-derived xenograft tumor growth in vivo

**Fig. 6:**
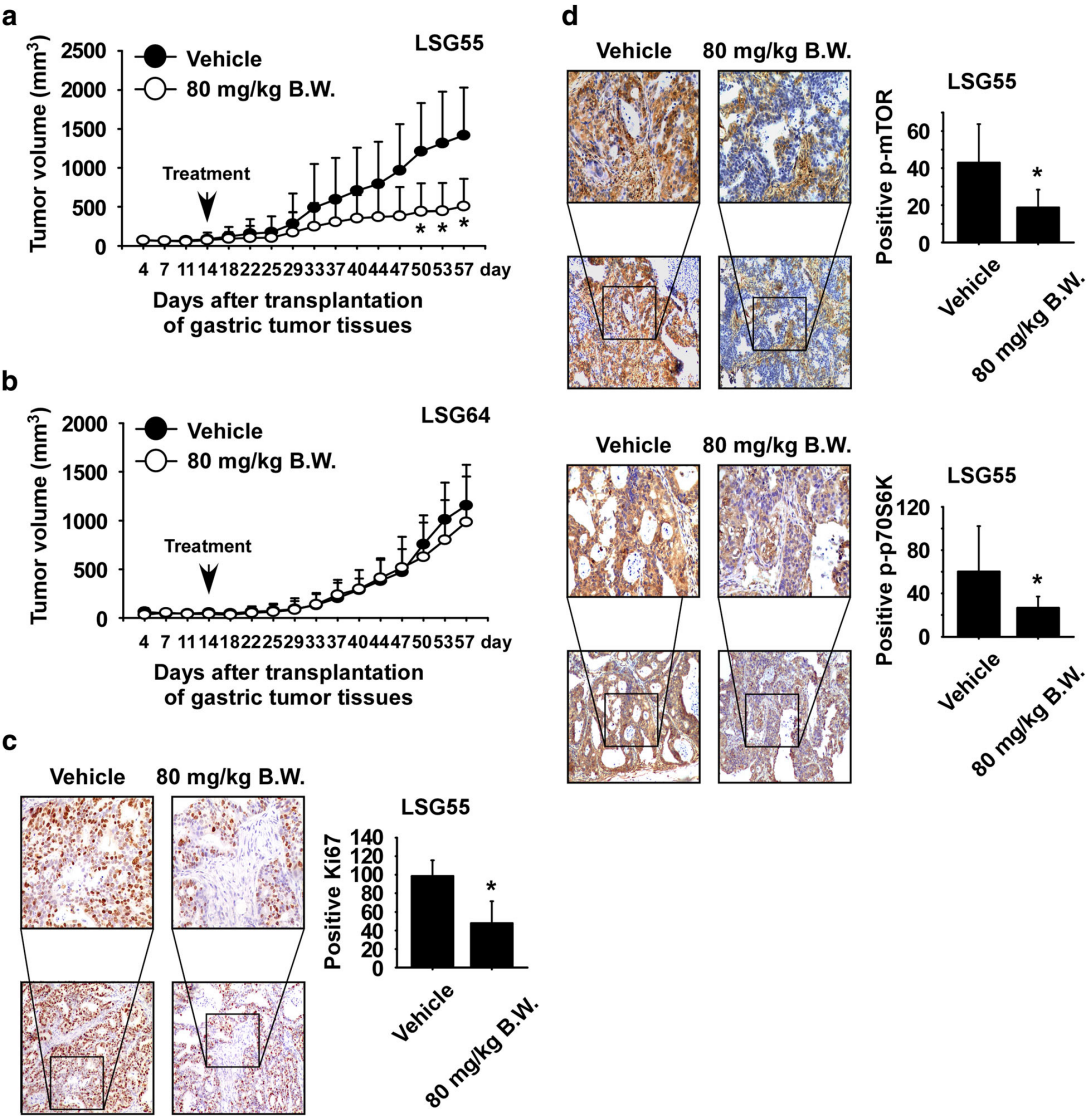
2,6-DMBQ reduces gastric cancer patient-derived xenograft tumor growth in vivo. Mice were divided into 2 groups to assess the effect of 2,6-DMBQ on gastric cancer patient-derived xenograft tumor growth. Groups are as follows: 1) vehicle group or 2) group treated with 80 mg/kg of 2,6-DMBQ. Tumor-bearing mice were orally administered (by gavage) 2,6-DMBQ or vehicle once a day Monday through Friday for 43 days. Tumor volumes were measured on the days indicated. The effect of 2,6-DMBQ on gastric tumor growth in (**a**) LSG55 or (**b**) LSG64 gastric PDX tissues. The asterisk (*) indicates a significant difference between tumors from untreated or treated mice as determined by t test (*p* < 0.05). **c** Effect of 2,6-DMBQ on Ki-67 expression. Treated or untreated groups of tumor tissues were stained with antibodies to detect Ki-67. **d** Effect of 2,6-DMBQ on the mTOR signaling pathway. Treated or untreated groups of tumor tissues were stained with antibodies to detect phosphorylated mTOR and phosphorylated p70S6K. For **c** and **d**, the number of Ki-67, phosphorylated mTOR or phosphorylated p70S6K-stained cells was counted from immunohistochemistry results (n = 6). All data are shown as means ± S.E. of values obtained from the experiment groups. The asterisk (*) indicates a significant difference between tumors from untreated or treated mice as determined by t test (*p* < 0.05)

To examine the toxicity of 2,6-DMBQ in vivo, mice were orally administrated 2,6-DMBQ at 20, 50, or 80 mg/kg or vehicle for 2 weeks, and then blood samples from each group mice were collected and analyzed. The results indicated that the activity of alanine transaminase (ALT) and aspartate transaminase (AST) were not significantly changed in mice treated with 2,6-DMBQ at 20, 50 or 80 mg/kg compared with the vehicle-treated group (Supplemental Fig. 3a, b). Therefore, we used 80 mg/kg 2,6-DMBQ for the PDX study. To investigate whether 2,6-DMBQ could reduce gastric tumor growth in vivo, we established gastric cancer patient-derived xenografts in mice. To determine the expression of phosphorylated mTOR protein, gastric tumor tissues were analyzed by Western blotting. Tissues expressing high (LSG55) or low (LSG64) levels of phosphorylated mTOR (Supplemental Fig. 4 and Supplemental Table 1) were implanted into the back of the neck of SCID mice. Mice were orally fed vehicle or 80 mg/kg 2,6-DMBQ 5 times a week over a period of 43 days. The results showed that administration of 2,6-DMBQ significantly reduced the volume of LSG55 gastric tumors relative to the vehicle-treated group (Fig. 6a; *p* < 0.05). In contrast, 2,6-DMBQ had little effect on the growth of LSG64 tumors (Fig. 6b). These results indicated that growth of LSG55 PDX tissue (high level of phosphorylated mTOR) was susceptible to 2,6-DMBQ’s effect compared to LSG64 PDX tissue (low level of phosphorylated mTOR) growth (Fig. 6a, b). 2,6-DMBQ-treated mice exhibited no significant loss of body weight compared with the vehicle-treated group (Supplemental Fig. 5a, b). To evaluate the potential toxic effect of 2,6-DMBQ on the body, the liver, spleen and kidney tissue extracted from vehicle-treated and 2,6-DMBQ-treated mice were stained with hematoxylin and eosin (H&E). Results indicated no distinct morphological changes in the 2,6-DMBQ-treated group compared to vehicle-treated group mice (Supplemental Fig. 6a-c). We next investigated whether 2,6-DMBQ affects the expression of the tumor proliferation marker Ki-67 by using immunohistochemistry. Results indicated that the expression of Ki-67 was significantly decreased in LSG55 tissues by 2,6-DMBQ treatment (Fig. 6c). To validate whether 2,6-DMBQ could suppress the mTOR signaling pathway, PDX tumor tissues were analyzed by immunohistochemistry. Results indicated that the expression of phosphorylated mTOR (Fig. 6d, *upper panel*) and phosphorylated p70S6K (Fig. 6d, *lower panel*) was strongly reduced in the 2,6-DMBQ-treated group.

## Discussion

Dietary intake of quinones have been reported to show cancer prevention through inhibitory effects on cell proliferation and tumor development [26]. 2,6-DMBQ is a benzoquinone compound that is isolated from sourdough fermentation of wheat germ. Recently, 2,6-DMBQ has been reported to possess cancer prevention properties against TPA-induced skin carcinogenesis [27]. However, the molecular targets of 2,6-DMBQ and its potential therapeutic effect have not been investigated in cancer. In this study, we report that 2,6-DMBQ reduces the growth of gastric cancer by targeting mTOR in vitro and in vivo.

The results of signaling pathway (Fig. 3a, b) and in vitro kinase assay (Fig. 3c) strongly support that 2,6-DMBQ is a potent mTOR protein kinase inhibitor and can reduce mTOR signaling pathway in gastric cancer cells. Additionally, the results of our cancer-related kinase screening showed that 2,6-DMBQ at 10 μM reduced about 30% of the PKCα activity (Supplemental Fig. 2). Therefore, we next examined whether 2,6-DMBQ could affect growth of gastric cancer cells through targeting PKCα. We first investigated the effect of PKCα inhibitor (CMPD101) on growth of gastric cancer cells. The results showed that PKCα inhibitor significantly reduced growth of gastric cancer cells (Supplemental Fig. 7a, b). We next assessed the effect of 2,6-DMBQ combined with PKCα inhibitor on gastric cancer cell growth. Cells were treated with PKCα inhibitor combined with or without 2,6-DMBQ. Results indicated that PKCα inhibitor-treated cells were not resistant to 2,6-DMBQ’s effect on cell growth compared to 2,6-DMBQ-treated cells (Supplemental Fig. 7c, d). Therefore, we suggest that it is highly likely that 2,6-DMBQ preferentially targets mTOR as opposed to PKCα. However, the result of the anticancer effect upon treatment with 2,6-DMBQ in cells with low mTOR expression (*shmTOR #4)* suggested that 20 μM of 2,6-DMBQ still reduced cell growth (Fig. 5a, b). It is possible there are other molecular targets of 2,6-DMBQ. Therefore, additional studies are planned to further characterize 2,6-DMBQ in identifying additional potential molecular targets.

mTOR signaling plays an important role in G1 to S phase cell cycle transition through regulation of cyclin D1 and c-myc expression [28], and inhibition of mTOR activity by an mTOR inhibitor induced G1 phase cell cycle arrest [29]. Based on the results of cell cycle and cell cycle marker proteins (Fig. 1d, e), we suggest that the reduction of mTOR activity by 2,6-DMBQ treatment may induce G1 phase cell cycle arrest and reduce the expression of cyclin D1 and cyclin D3.

Although many anticancer reagents have shown favorable tumor responses in preclinical studies, only 5% of anticancer drugs developed have been approved by the Food and Drug Administration (FDA) [30, 31]. This is due to a number of reasons, including the development of resistance conferred by tumor heterogeneity as well as human stromal microenvironmental conditions [32]. Therefore, to overcome low clinical efficacy, researchers established the patient-derived xenograft (PDX) model to screen potential candidate drugs [33]. We first investigated the antitumor effects of 2,6-DMBQ on gastric cancer PDX models and the results showed that 2,6-DMBQ significantly reduced gastric tumor growth by inhibiting the mTOR/p70S6K signaling pathway (Fig. 6a, d).

Previously, phosphorylated mTOR was found to be significantly over-expressed and correlated with various clinical and pathologic parameters in patients with gastric cancer [34, 35]. Additionally, the mTOR signaling pathway is positively correlated with Ki-67 expression [36–38] and rapamycin was found to inhibit Ki-67 expression in patients with glioblastoma [39]. Therefore, we examined whether 2,6-DMBQ could reduce the expression of Ki-67 in gastric cancer PDX tissues. We found that the expression of Ki-67, phosphorylated mTOR and phosphorylated p70S6K was significantly decreased in the 2,6-DMBQ-treated group compared to the vehicle-treated group (Fig. 6c, d). Therefore, reducing mTOR signaling by an inhibitor could provide antineoplastic effects for treatment of gastric cancer.

In conclusion, our findings demonstrate that 2,6-DMBQ is a potent mTOR inhibitor that reduces growth of gastric cancer. These findings could be useful for treating gastric cancers.

## Conclusions

Fermented wheat germ extract (FWGE) has been reported to possess various pharmacological effects. However, the anticancer activity of FWGE and its molecular mechanism(s) against gastric cancer have not been characterized. Here, we present novel results suggesting that 2,6-DMBQ, a major compound in FWGE, is a novel mTOR inhibitor that exhibits anticancer properties in vitro and in vivo which make it a potential candidate that may be useful in treating gastric cancer.

## Supplementary information

**Additional file 1: Supplemental Figure 1.** IC_50_ value of 2,6-DMBQ in gastric cancer cells. Effect of 2,6-DMBQ on the growth of HGC27 (a) or AGS (b) gastric cancer cells. Cells were treated with 2,6-DMBQ at various concentrations and then incubated for 48 h before the growth was determined by MTT assay. For a and b, data are shown as mean ± S.E. of values obtained from 3 independent experiments.

**Additional file 2: Supplemental Figure 2.** Screening of the effect of 2,6-DMBQ on various kinases. The effect of 2,6-DMBQ on the kinase activity of ABL, AMPKα1, AURKA, B-RAF, CDK2/CCNE, CHEK1, EGFR, FAK, FGFR1, FYN, GSK3β, MAPK1, MEK1, MET, PDK1, PKBα, PKCα, p70S6K, RSK2, SAPK2α, SRC, TAK1 or TBK1 was determined using active protein kinases and the specific substrates for each kinase. Data are shown as mean ± S. D of values.

**Additional file 3: Supplemental Figure 3.** 2,6-DMBQ has no toxicity in vivo. The effect of 2,6-DMBQ on the activity of AST (a) or ALT (b) was accessed. Mice were orally administered 2,6-DMBQ (20, 50, or 80 mg/kg B.W.) or vehicle for 2 weeks before blood was collected. AST and ALT activity were calculated from 2,6-DMBQ -treated or vehicle-treated mice. All data are shown as mean ± S.E. of values obtained from each group (n = 4).

**Additional file 4: Supplemental Figure 4.** The expression of phosphorylated mTOR and p70S6K in gastric PDX tissues. The expression of phosphorylated mTOR, −p70S6K and β-Actin in LSG55 and LSG64 gastric PDX tissues was accessed by Western Blot.

**Additional file 5.**

**Additional file 6: Supplemental Figure 5..** Effect of 2,6-DMBQ on mouse body weight. Mice were orally administrated vehicle or 2,6-DMBQ at 80 mg/kg 5 times a week for 43 days by the gavage method. (a, b) Effect of 2,6-DMBQ on mouse body weight. Body weight from treated or untreated groups of mice were obtained once a week over the timespan of 57 days. For a and b, data are shown as means ± S.E. of values obtained from experiments.

**Additional file 7: Supplemental Figure 6.** 2,6-DMBQ has low toxicity in vivo. Immunohistochemistry analysis of liver (a), kidney (b) and spleen (c) tissues. Treated or untreated groups of liver, kidney or spleen tissues were stained with H&E.

**Additional file 8: Supplemental Figure 7.** Effect of PKCα inhibitor combined with 2,6-DMBQ on growth of gastric cancer cells. (a, b) Effect of PKCα inhibitor on growth of gastric cancer cells. Cells were treated with various concentrations of PKCα inhibitor for 48 h and cell growth was assessed by MTT assay. (c, d) Effect of PKCα inhibitor combined with 2,6-DMBQ on growth of gastric cancer cells. Cells were treated with or without PKCα inhibitor and various concentration of 2,6-DMBQ for 48 h and cell growth was assessed by MTT assay. All data are shown as mean ± S.D. of values from 3 independent experiments and the asterisk (*) indicates a significant difference (*p* < 0.05).

## Data Availability

Supplemental Figures (1, 2, 3, 4, 5, 6 and 7), supplemental Table (1) and associated figure legends are provided as supplemental material and are available online with the paper.

## Abbreviations

mTOR: Mammalian target of rapamycin
GC: Gastric cancer
p70S6K: p70 ribosomal S6 kinase
RAPTOR: Regulatory-associated protein of mTOR
RICTOR: Rapamycin-insensitive companion of mTOR
EGFR: Epidermal growth factor receptor
RSK: Ribosomal protein S6 kinase
AKT: V-Akt murine thymoma viral oncogene homolog
PDX: Patient-derived xenograft
