# Supplementary figures and images for: 2,6-DMBQ is a novel mTOR inhibitor that reduces gastric cancer growth in vitro and in vivo
Source: J Exp Clin Cancer Res. 2020 Jun 9;39:107. doi: 10.1186/s13046-020-01608-9 (PMC7285595; doi:10.1186/s13046-020-01608-9)

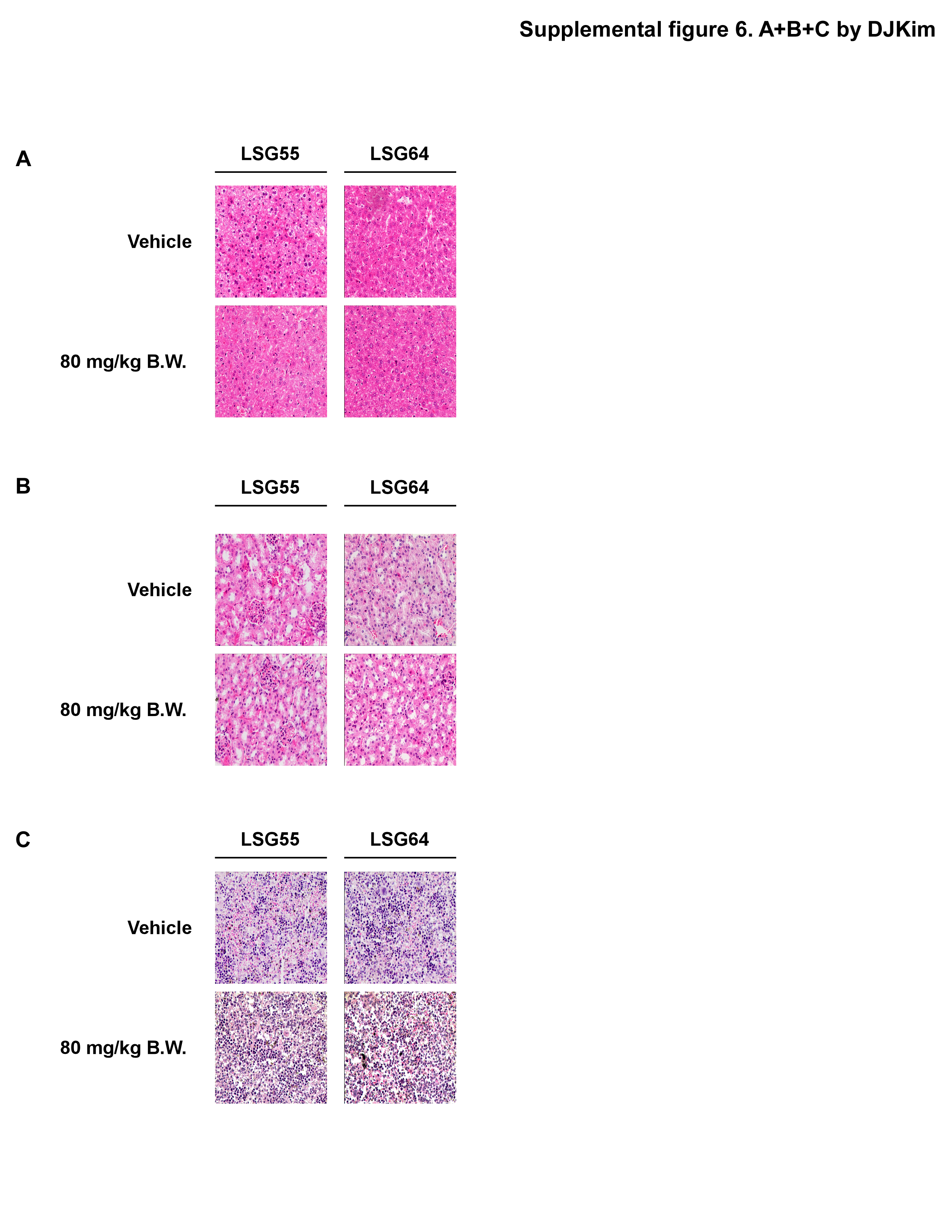

Supplement: Supplementary file 7 — Additional file 7: Supplemental Figure 6. 2,6-DMBQ has low toxicity in vivo. Immunohistochemistry analysis of liver (a), kidney (b) and spleen (c) tissues. Treated or untreated groups of liver, kidney or spleen tissues were stained with H&E. [file 13046_2020_1608_MOESM7_ESM.tif]
